# A survey of knowledge, attitudes and practice of emergency contraception among university students in Cameroon

**DOI:** 10.1186/1471-227X-7-7

**Published:** 2007-07-17

**Authors:** Eugene J Kongnyuy, Pius Ngassa, Nelson Fomulu, Charles Shey Wiysonge, Luc Kouam, Anderson S Doh

**Affiliations:** 1 Child and Reproductive Health Group, Liverpool School of Tropical Medicine, UK; 2 Department of Obstetrics and Gynaecology, Faculty of Medicine and Biomedical Sciences, University of Yaounde I, Cameroon; 3 South African Cochrane Centre, Medical Research Council, Cape Town, South Africa

## Abstract

**Background:**

Unsafe abortion is a major public health problem in low-and-middle income countries. Young and unmarried women constitute a high risk group for unsafe abortions. It has been estimated that widespread use of emergency contraception may significantly reduce the number of abortion-related morbidity and mortality. The purpose of this study was to evaluate the knowledge, attitudes and experiences on emergency contraceptive pills by the university students in Cameroon in order to develop and refine a national health programme for reducing unwanted pregnancies and their associated morbidity and mortality.

**Methods:**

A convenient sample of 700 students of the University of Buea (Cameroon) was selected for the study. Data was collected by a self-administered, anonymous and pre-tested questionnaire.

**Results:**

The response rate was 94.9% (664/700). General level of awareness of emergency contraceptive pills was 63.0% (418/664). However, knowledge of the general features of emergency contraceptive pills was low and misinformation was high among these students. Knowledge differed according to the source of information: informal source was associated with misinformation, while medical and informational sources were associated with better knowledge. Although the students generally had positive attitudes regarding emergency contraceptive pills, up to 65.0% (465/664) believed that emergency contraceptive pills were unsafe. Those with adequate knowledge generally showed favourable attitudes with regards to emergency contraceptive pills (Mann-Whitney U = 2592.5, p = 0.000). Forty-nine students (7.4%) had used emergency contraceptive pills themselves or had a partner who had used them.

**Conclusion:**

Awareness of emergency contraception pills by Cameroonian students is low and the method is still underused. Strategies to promote use of emergency contraception should be focused on spreading accurate information through medical and informational sources, which have been found to be reliable and associated with good knowledge on emergency contraceptive pills.

## Background

Emergency contraception (EC) is contraception administered after unprotected intercourse to prevent pregnancy. It is also known as "post-coital contraception", and is less effective than regular contraception. EC is intended for occasional or emergency use only and not as a regular contraception. It is associated with a failure rate of 0.2% to 3% [1]. There are various methods of emergency contraception. They include hormonal contraceptive pills (also called morning-after pills), intrauterine contraceptive devices and mifepristone. Formerly, emergency contraceptive pills (ECPs) were thought to be effective only within 72 hours, but recent studies have confirmed that they are effective for up to 120 hours [2,3]. A Cochrane review concluded that levonorgestrel 1.5 mg (two split doses or a single dose) and low and mid-doses (25–50 mg) of mifepristone were highly effective with an acceptable side-effect profile [4]. A single dose simplifies the use of levonorgestrel for emergency contraception without an increase in side-effects.

Situations of unprotected intercourse that demand the use of emergency contraception include failure of barrier methods such as slippage, breakage or misuse of condom, sexual assaults, failed coitus interruptus, two or more consecutive missed oral contraceptive pills, or simply because intercourse was unexpected and therefore contraception had not been used [2]. Although emergency contraception has been available in many countries for the last three decades, it remains relatively unknown and underused in those countries [5].

The World Health Organization estimates that 84 million unwanted pregnancies occur annually worldwide [6]. Averagely, 46 million abortions take place every year, out of which 20 million are performed under unsafe conditions [6,7]. Seventy thousand women die yearly as a consequence of unsafe abortion, while five million suffer permanent or temporary disability [6-9]. Approximately 13% of pregnancy-related mortality worldwide is due to unsafe abortions and the majority of these deaths (and morbidity) occur in low-and-middle income countries [11]. An important proportion of maternal deaths worldwide is attributable to induced unsafe abortion: Asia (20–25%), Africa (30–50%) and Russia (25–30%) [10,11].

The consequences of unplanned pregnancies in Cameroon are multiple. They include discontinuation of school, unsafe illegal abortions and their risk of very serious morbidity and mortality. Abortion is not legalized in Cameroon and therefore the exact number of abortion cases is not known [12]. In 2000, the estimated maternal mortality ratio attributed to unsafe abortion in Cameroon was 90 to 100,000 live births [13]. Unsafe abortion is responsible for 27.4% to 34.6% of maternal deaths in Cameroon [14-16]. The most affected groups are students, single and nulliparous young girls [15,16]. It has been estimated that widespread use of emergency contraception may reduce the number of abortions in the United States by more than 1 million annually [17]. Therefore, emergency contraception could be a potential strategy to reduce the incidence of unwanted pregnancies and unsafe abortions in Cameroon.

In recent years, many low-and-middle income countries have supported the use of emergency contraception. In Cameroon, ECPs are available in two forms: (a) levonorgestrel 0.75 mg, a progestin-only pill, and (b) certain brands of combined oral contraceptives given in slightly higher doses. Both forms can be obtained from family planning clinics and pharmacies without medical prescription. The major factor limiting the use of ECPs in Cameroon may therefore be inadequate information about their effectiveness and availability or unfavourable opinions about their safety due to misinformation.

Research on the knowledge, attitudes, and practices of the public regarding ECPs may help to inform policy-makers in Cameroon. Unfortunately little or no research has been conducted in this area in the country. Therefore the aim of our study is to evaluate the extent of the knowledge, attitudes and practice of ECPs in Cameroon with the view to identifying a plausible strategy for reducing unwanted pregnancies and the associated morbidity and mortality. Our study focuses on knowledge, use and attitudes of university students because the risk of unplanned pregnancy among young women, particularly students, in Cameroon is high [14]. We hope that our study will provide baseline data to assist policy makers in developing appropriate evidence-based strategies to promote the use of emergency contraceptive pills in Cameroon.

## Methods

### Study design and setting

This cross-sectional survey was carried out in March 2005 at the University of Buea, which is one of the six state universities in Cameroon and is the only state university in the English-speaking part of the country. The university has five faculties, namely, Management and Social Sciences, Health Sciences, Sciences, Arts, and Education. The student population of University of Buea is approximately 7000, and it has an infirmary which does not offer family planning services. However, students have access to family planning services at the Buea District Hospital (situated at less than 1 km from the university campus), several nearby public health centres, and private pharmacies and medical clinics. The study was approved by the Ethics Committee of the University of Yaounde I. In addition, authorisation was obtained from the appropriate authority of the University of Buea prior to the day of the survey,

### Study population and sampling

A convenience sampling method was used. All students of the university were eligible for the survey. However, only students who were present in the university campus at the time the study was conducted actually participated in the survey.

On the day of administration of the questionnaire, students who were found in the university campus were requested to gather in two amphitheatres of the University. To reduce the non-respondent rate, the information was not disclosed until all students had gathered in the two lecture halls. They were then informed of the survey, its objectives and procedures, and assured that the information collected would be treated as confidential and used only for research purposes. Students who gave their verbal informed consent were provided with the four-page, anonymous, self-administered questionnaire. Students were well spaced out to avoid communication among them during the exercise. They were also asked to request for clarification if any item in the questionnaire was not clear. Students were not required to identify themselves by writing their names on the questionnaire and confidentiality was emphasized. The exercise took about an hour. Each amphitheatre had two baskets in which students were asked to put the completed questionnaire before leaving the halls.

### Study instrument

We used a four-page, anonymous, self-administered questionnaire for data collection. The questionnaire had both closed and open-ended questions. It was composed of five parts. The first part, which was the preamble, contained the following definition of ECPs: "Emergency contraceptive pills are also called morning-after pills. They are used to prevent pregnancies after unprotected sexual intercourse or after condom breakage during a suspected fertile period." The second part contained information on the demographic characteristics of the study participants. The third part assessed the knowledge of students about emergency contraceptive pills; the fourth part evaluated their attitudes while the fifth part was concerned with their practices as regards ECPs.

The questionnaire was initially designed taking into consideration similar surveys that have been carried out in other countries [18]. We modified the original questionnaire to suit our context after pre-testing it among the students of the University of Yaounde I. The pretest questionnaire was distributed to 20 students who all completed and returned. The completed questionnaires were then studied to identify major issues that needed amendment. As a result, some questions were rephrased and the question "ECP is a method of early abortion – yes or no" was added to the questionnaire after realizing during the pre-testing exercise that many students believed that ECP was a method of abortion.

We determined the knowledge about ECPs using four multiple-choice questions. The four questions to evaluate the level of knowledge about ECPs were: (1) "which of these is an emergency contraceptive pill?", (2) "what is the maximum acceptable time after sex for a woman to take the ECP?", (3) "ECP is a method of early abortion", (4) "when taken early, ECPs can prevent sexually transmitted infections". Each correct question corresponded to 1 point, and so there was a total of 4 points for the four questions. Students were considered to have adequate knowledge if they scored 3 or 4 out of 4. They were considered to have inadequate knowledge if they scored between 0, 1 and 2 out of 4.

The students' attitudes were measured using four items rated on a four-point Likert scale as (1) strongly disagree, (2) disagree, (3) agree and (4) strongly agree. The four items were: (a) "I would use ECP if I have unprotected intercourse during the unsafe period", (b) "The ECP is safe for its users", (c) "I would recommend ECPs to a friend" and (d) "Providing ECPs would discourage consistent use of condom". Using this four-point scale for 4 questions, we arbitrarily set the maximum score for each respondent at 16 and the minimum at 4. We decided that a high score was indicative of positive attitude while a low score would be indicative of a negative attitude.

Part five of the questionnaire required the students to state their prior experience with ECPs and associated sexual risk practices. It consisted of 10 structured questions.

### Statistical analyses

The data was entered and analyzed with the Statistical Package for Social Sciences (SPSS) software programme (version 12.0, Chicago, IL, USA). For descriptive statistics results were expressed in terms of proportions or percentages and for analytical statistics odds ratios were used to examine the relation between variables. The Mann-Whitney test was used to examine the relationship between knowledge and attitude.

## Results

### Participation rate

Seven hundred students were approached and 664 accepted to participate in the study. All the 664 students completed and returned the questionnaires, giving a total participation rate of 94.9%.

### Socio-demographic characteristics

Table 1 presents the socio-demographic characteristics of the study participants. The mean age of the respondents was 21 (range 16 to 40). Three hundred and eighty respondents (57.2%) were males and 636 respondents (95.8%) were single.

**Table 1:** Socio-demographic characteristics of the respondents, by sex.

**Characteristic**	**Male (n = 380) N (%)**	**Female (n = 284) N (%)**	**Total (n = 664) N (%)**
Mean age (range)	21.3 (17–40)	20.7 (16–28)	21.0 (16–40)
Age groups (years)			
16–19	80 (21.1)	70 (24.6)	150 (22.6)
20–24	220 (57.9)	176 (62.0)	396 (59.6)
25–29	26 (6.8)	8 (2.8)	34 (5.1)
30+	4 (1.2)	0 (0.0)	4 (0.6)
Missing	50 (13.2)	30 (10.6)	80 (12.0)
Marital status			
Single	372 (98.0)	264 (93.0)	636 (95.8)
Married	2 (0.5)	16 (5.6)	18 (2.7)
Missing	6 (1.6)	4 (10.4)	10 (1.5)
Religious groups			
Christianity	355 (93.4)	269 (94.7)	624 (94.0)
Islam	4 (1.1)	3 (1.1)	7 (1.1)
Atheism	8 (2.1)	5 (1.8)	13 (2.0)
Other groups	13 (3.4)	7 (2.5)	20 (3.0)
Level in University			
First	120 (31.6)	95 (33.5)	215 (32.4)
Second	128 (33.7)	104 (36.6)	232 (34.9)
Third	117 (30.8)	75 (26.4)	192 (28.9)
Postgraduate	15 (3.9)	10 (3.5)	25 (3.8)

### Knowledge of ECPs

Four hundred and eighteen students (63.0%) reported that they had heard of "ECPs" or "morning-after pills" before. Of the 380 male respondents and 284 female respondents, 240 (63.2%) and 178 (62.7%) respectively reported prior knowledge of EC.

A majority of respondents in this study had knowledge from friends and family members. The sources were as follows: 291 (69.6%) from friends and family members, 83 (19.9%) from various health personnel (doctors, nurses and pharmacists) and 44 (10.5%) from audio-visual media (television, radio, internet and books).

It can be seen from table 2 that only 32 participants (4.8%) could identify levonorgestrel (Norlevo^®^) as an emergency contraceptive method, and 130 (19.6%) thought Synergon^® ^was an ECP. Thirty eight students (5.7%) knew that the first dose of ECPs could be taken up to 72–120 hours after unprotected sex. Overall, knowledge of what constitutes EC was poor.

**Table 2:** Students' knowledge about emergency contraceptive pills

**Item**	**N**	**%**
Which of these is an ECP?		
Synergon	130	19.6
Norlevo	32	4.8
Orgametril	16	2.4
Duphaston	9	1.4
Don't know	477	71.8
What is the maximum acceptable time after sex for a woman to take ECP?		
12–18 hours	36	5.4
24–48 hours	56	8.4
72–120 hours	38	5.7
124–160 hours	25	3.8
Don't know	509	76.7
Emergency contraceptive pill is a method of early abortion		
No	182	27.4
Yes	340	51.2
Don't know	142	21.4
When taken early, ECP can prevent STIs		
No	448	67.2
Yes	118	17.8
Don't know	98	14.8

STIs = sexually transmitted infections

ECP = Emergency contraceptive pill

Table 3 compares the characteristics of respondents with adequate and inadequate knowledge. There were 48 respondents (7.2%) who demonstrated adequate knowledge of ECPs. Factors significantly associated with adequate knowledge were female sex (*p *= 0.005) and previous ECP use (*p *< 0.001).

**Table 3:** Comparison of selected characteristics of respondents with adequate and inadequate knowledge^a^

	**KNOWLEDGE**					
			
**VARIABLES**	**Adequate**		**Inadequate**		**ODDS RATIO (95% CI)**	**P-VALUE**
	**N**	**%**	**N**	**%**		
Sex (female)	30	62.5	254	41.2	2.38 (1.25–4.62)	0.005
Previous use of ECP	11	22.4	38	6.2	4.52 (1.92–9.93)	<0.001
Age ≤ 19 years	14	29.2	136	22.1	1.45 (0.74–2.76)	0.246
Married	2	4.2	16	2.6	1.63 (0.25–6.42)	0.257
Religion (Christians)	46	95.8	578	93.8	1.51 (0.41–9.58)	0.641
Sexually active	42	87.5	547	88.8	0.88 (0.38–2.36)	0.726
Has been pregnant or impregnated someone	4	8.3	47	7.5	1.10 (0.32–2.98)	0.675

^a ^Criteria for adequate knowledge are given in the methodology

ECP = Emergency contraceptive pill

It would appear that respondents who obtained knowledge from friends and family members were more likely to have inadequate knowledge of EC than respondents who had prior knowledge from audio-visual media (96.6% versus 65.9%).

### Attitudes towards ECPs

The attitude of students towards ECPs is shown in Table 4. In the first item, 464 students (69.9%) either agreed or strongly agreed that they would use ECPs in the future if need arose, while the rest of the 200 students (30.1%) either disagreed or strongly disagreed. Overall, there was a positive attitude indicating a strong tendency of use of EC in the future by respondents.

**Table 4:** Students' attitudes towards emergency contraceptive pills

**Item**	**Strongly disagree**		**Disagree**		**Agree**		**Strongly agree**	
	**N**	**%**	**N**	**%**	**N**	**%**	**N**	**%**
I would use ECP if I have unprotected intercourse during the unsafe period	66	9.9	134	20.5	305	45.9	159	23.9
I would recommend ECPs to a friend	80	12.0	133	20.0	292	44.0	159	23.9
The ECP is safe for its users	281	42.3	184	22.7	139	20.9	60	9.0
Providing ECPs would discourage consistent use of condom	108	16.3	301	45.3	200	30.1	55	8.3

ECPs = Emernegency contraceptive pills

### Relationship between knowledge and attitudes

The relationship between knowledge and attitudes of respondents was investigated using the Mann-Whitney test. Knowledge was considered a categorical variable (adequate and inadequate) while each item of attitude was rated so that "strongly disagree" was 1 point and "strongly agree" was 4 points. The minimum overall score for each respondent was 4 and the maximum was 16. There was statistically significant association between knowledge and attitudes towards ECP. Those with adequate knowledge generally showed favourable attitudes with regards to ECPs (Mann-Whitney U = 2592.5, p = 0.000).

### EC practice among respondents

A total of 49 out of 664 respondents (7.4%) reported that they or their partners had previously used ECPs. The socio-demographic characteristics of respondents are shown in table 5. As concerns gender, 12.7% (36/284) of the female respondents compared to 3.4% (13/380) of male respondents were previous ECP users. Of the 284 female respondents, 25 (8.8%) declared that they had aborted before. Of the 25 cases of self-reported history of abortion, 2 (5.6%) were previous ECP users and 23 (92.0%) were non-users.

**Table 5:** Socio-demographic characteristics of respondents, by ECP user status

**Parameter**	**Previous ECP user (n = 49) N (%)**	**ECP non-user (n = 615) N (%)**	**OR or MD (95% CI)**	**p-value**
			**MD (95% CI)**	
Mean age (range)	21.7 (18–25)	20.9 (16–40)	-0.80 (-0.07, 1.67)	0.073
Mean age at first sexual relation (range)	17.2 (16–20)	17.3 (14–23)	-0.10 (-1.26, 1.06)	0.866
Sex			**OR (95% CI)**	
Male	13 (26.5)	367 (59.7)	1	0.103
Female	36 (74.5)	248 (40.3)	0.55 (0.25–1.17)	
Marital status*				
Married (n = 18)	2 (4.1)	16 (2.6)	1	0.512
Single (n = 636)	47 (95.9)	589 (97.4)	1.57 (0.24–6.16)	
Sexually active				
Yes	49 (100.0)	540 (87.8)	1	0.004
No	0 (0.0)	75 (12.2)	infinity (2 to infinity)	
Had been pregnant or impregnated someone				
Yes	4 (8.2)	47 (7.6)	1	0.678
No	45 (91.8)	568 (92.4)	1.07 (0.32–2.90)	
Has aborted (females only)*				
Yes (n = 25)	2 (5.6)	23 (9.4)	1	0.648
No (n = 255)	34 (94.4)	221 (90.6)	0.57 (0.09–2.19)	

* All respondents with missing responses were excluded from the analyses

ECP = Emergency contraceptive pill; OR = odds ratio; MD = difference between the two means

## Discussion

The awareness of ECPs among students of the University of Buea was 63%. This level of awareness was higher than the level found among university students in Kenya (39%) and Ghana (43.2%) [19,20]. It was however lower than among university students in the USA (86%) and Jamaica (84%) [21,22].

Most students lacked adequate knowledge about the general features of ECPs. Knowledge about the correct time for taking ECPs after unprotected sex was low (5.7%). This finding is lower than 11.3% reported in Ghana [20]. More than half of the respondents (51.2%) thought ECP was a form of abortion, compared to 25.8% of university students in Ghana and 49% of nursing students in Kenya [19,20]. In a similar survey in South Korea, students showed general lack of knowledge about ECPs and with a lot of misconceptions about their safety [21].

A large number of students wrongly believed that Synergon was an EC method. Synergon is dedicated product made up of a combination of progesterone and oestrone in an injectable form. It is used to induce withdrawal bleeding in cases of non-gravid amenorrhoea. In Cameroon, synergon is widely used among students as an abortifacient and is usually administered clandestinely by nurses when a woman presents with amenorrhoea. Such an attitude stemmed from the fact that this product induces withdrawal bleeding, so that some cases of amenorrhoea falsely believed to be due pregnancy are resolved by one or a few intramuscular injections of Synergon. We are not aware of similar findings reported from other countries. Synergon is not produced by the manufacturer as an abortifacient; how it came to be associated with abortion in Cameroon is not known. It is also not known whether it actually induces abortion or it only induces withdrawal bleeding in women with non-gravid amenorrhoea.

The most important sources of information on ECPs for these students were informal networks such as friends and family members. Similar findings have been reported by other authors [20,22,23].

Unfortunately, the informal network, which was the largest source of information on ECPs for the students turned out to be unreliable. Informal source was associated with misinformation, while medical and informational sources were associated with better knowledge. Our findings differ from those of Sorhaindo et al who reported that, although informal networks were the main sources of information on ECPs in Jamaica, most students had correct knowledge about the general characteristics of ECPs [22]. They therefore suggested that strategies to promote ECP use could take advantage of the informal sources, which were already providing accurate knowledge to the Jamaican students.

Although our students generally held favourable opinions about ECPs, most of them believed that ECPs were unsafe for their users. Similar findings have been reported by other authors [19,20,22]. Two hundred and fifty five participants (38.4%) thought providing ECPs would discourage the consistent use of condoms compared to 53.4% of students in Ghana [20]. Prior to this study, we held a focus group discussion with students of the University of Yaounde I, in Cameroon's political capital city. The students were unanimous that the principal objective of condom use was to prevent HIV and so the availability of ECPs could not discourage the use of condoms; if the users understand that ECPs do not prevent HIV infection.

Forty-nine students (7.4%) reported that they or their partners had used ECPs before, compared to 10% of students in Jamaica and 7.5% of nursing students in Kenya [19,23]. This may be because most of the students in this study used ECPs because either a contraceptive method was not used during sexual intercourse or because of condom breakage or slippage.

This article is the first of its type to look at emergency contraception among students in Cameroon. The use of convenience sampling in this study is a limitation; as such a sample may not be representative of the target population. In addition, we cannot guarantee that students provided honest answers to the questions, since the survey involved a sensitive matter (i.e. sex).

## Conclusion

We conclude that awareness of ECPs by Cameroonian students is low; that the knowledge about the general features of ECPs is low and misinformation is high among these students; that although the students generally have positive attitudes regarding ECPs, most of them believe that ECPs are unsafe for their users; and that, despite widespread availability of ECPs in Cameroon for several years, the method is still underused. We strongly recommend that strategies to promote ECP use be focused on spreading accurate information through information, education and communication in by medical personnel and through audio-visual media, which have been found to be reliable and associated with good knowledge on ECPs.

## Competing interests

The author(s) declare that they have no competing interests.

## Authors' contributions

EJ Kongnyuy and P Ngassa conceived and designed the study, and collected, analysed and interpreted the data; N Fomulu and CS Wiysonge critically revised the study protocol and the manuscript for important intellectual content; and L Kouam and AS Doh contributed intellectually to the final version of the manuscript. All authors read and approved the final version of the manuscript.

## Pre-publication history

The pre-publication history for this paper can be accessed here:


